# Unilateral biportal endoscopy for the treatment of adjacent segment disease after lumbar fusion in elderly patients: a matched comparison study

**DOI:** 10.3389/fsurg.2025.1723807

**Published:** 2025-11-24

**Authors:** Hongwei Duan, Minghui Liang, Yu Xi, Ruiyuan Chen, Ning Fan, Tianyi Wang, Aobo Wang, Ziqian Ma, Lei Zang, Shuo Yuan

**Affiliations:** Department of Orthopedics, Beijing Chaoyang Hospital, Capital Medical University, Beijing, China

**Keywords:** adjacent segment disease, unilateral biportal endoscopy, lumbar spinal stenosis, minimally invasive surgery, clinical efficacy

## Abstract

**Background:**

Unilateral biportal endoscopy (UBE) is an important minimally invasive surgical treatment option for lumbar spinal stenosis (LSS). However, to our knowledge, no studies have focused on UBE for treating adjacent segment disease (ASD) after lumbar fusion. Thus, this study aimed to analyze the clinical efficacy of UBE for ASD patients, and further compare it with non-ASD patients.

**Methods:**

This retrospective study enrolled consecutive patients who underwent UBE decompression surgery for LSS between January 2022 and March 2024. According to the inclusion and exclusion criteria, 82 patients were divided into study group (42 ASD patients) and control group (42 non-ASD patients matched by sex, surgical level, and age). Surgical outcomes were evaluated using the visual analog scale (VAS) and Oswestry Disability Index (ODI) obtained preoperatively, 3 days postoperatively, and at 3- and 12-month follow-ups, along with the MacNab criteria, cross-sectional area of the dural sac, and incidence of complications.

**Results:**

The ASD and non-ASD groups enrolled 42 patients each. Except for the operative time (*P* < 0.001), no significant differences in baseline characteristics were observed between the two groups. Both groups showed significant improvement in VAS scores, ODI scores, and dural sac cross-sectional area after UBE surgery (*P* < 0.001), with sustained clinical efficacy throughout the follow-up period. Although the ASD group had significantly higher preoperative VAS scores for back pain than the non-ASD group (4.78 ± 1.41 vs. 4.02 ± 1.65, *P* = 0.027), no significant differences were found between the groups at any postoperative follow-up (3 days, 3 months, or 12 months). Based on the MacNab criteria, excellent or good outcomes were observed in 85.7% (36/42) of the patients in the ASD group and 88.1% (37/42) in the non-ASD group, with no significant difference. The incidence of complications was comparable between the two groups (*P* = 1.000), and no severe complications were observed.

**Conclusion:**

UBE demonstrated favorable clinical outcomes and safety in treating ASD patients. It may represent a promising minimally invasive option for elderly patients with multiple comorbidities who cannot tolerate revision surgery.

## 1 Introduction

Spinal fusion surgery is widely performed for treating degenerative spinal diseases, which effectively stabilizes the affected segments with satisfactory outcomes (1). However, the use of pedicle screws for fixation irreversibly restricts the motion of the fused segments (2, 3), accelerating degenerative changes and likely resulting in adjacent segment disease (ASD). ASD is defined as the development of new symptoms that are directly associated with radiographic degenerative changes in spinal segments adjacent to a prior fusion procedure (4). The incidence rates of ASD range from 5.2% to 18.5% (5). Mild-to-moderate ASD cases are typically managed conservatively, but surgical intervention is necessary when conservative treatment fails to relieve symptoms (6).

The traditional surgical treatment is open revision surgery. Notably, compared with other common degenerative spinal diseases, ASD presents unique challenges. ASD patients often exhibit poorer general health, including older age and more comorbidities, and more complex anatomical changes (7). These characteristics increase the difficulty of surgical treatment, emphasizing the need for approaches that minimize trauma and achieve effective outcomes. Compared with open revision surgery, minimally invasive spinal surgery offers advantages such as minimizing damage to paraspinal muscles, ligaments, and other soft tissues and facilitating faster recovery (6), which makes it suitable for treating ASD patients.

Unilateral biportal endoscopy (UBE), an advanced minimally invasive technique for spinal surgery, has been widely employed for the treatment of degenerative spinal diseases such as lumbar spinal stenosis (LSS) and lumbar disc herniation, with satisfactory outcomes (8, 9). With its flexible operating approach and familiar working field, UBE not only shares the advantages of other minimally invasive surgeries but also addresses more complex lesions and significantly shortens the surgical time (10–12). Accordingly, we hypothesize that UBE could be a viable alternative to open surgery for ASD, assuming the absence of significant segmental instability. However, there has been no dedicated clinical study systematically evaluating UBE for ASD patients. Therefore, this study aimed to evaluate the efficacy and safety of UBE in treating ASD, and including a matched non-ASD control group allows us to determine whether UBE maintains comparable efficacy and safety in the more complex anatomical setting of ASD.

## 2 Methods

### 2.1 Patient population

This study retrospectively analyzed consecutive patients who underwent UBE for LSS at our institution between January 1, 2022, and March 30, 2024. All patients were deemed suitable for UBE based on current indications. This retrospective study was approved by our hospital's ethics committee and conducted in accordance with the Declaration of Helsinki. The ethics committee waived the requirement for informed consent due to the retrospective nature of this study.

The inclusion criteria for the ASD group were as follows: (1) history of posterior lumbar fusion surgery for degenerative lumbar disease; (2) unilateral lower back, buttock, or leg pain (LP), or neurogenic claudication; (3) imaging-confirmed adjacent segment LSS correlating with symptoms; (4) absence of significant lumbar intervertebral instability on flexion-extension radiographs, defined as sagittal plane translation <3 mm and angular motion <10° between adjacent vertebrae; (5) persistent symptoms unresponsive to conservative treatment for ≥3 months; and (6) complete 12-month follow-up records. The exclusion criteria for the ASD group were as follows: (1) degenerative lumbar disease unrelated to the fused segments; (2) symptoms inconsistent with adjacent segment degeneration; (3) biportal symptoms; (4) prior surgery at the same lumbar level; (5) severe lumbar spinal stenosis with Schizas grade D; (6) other spinal pathologies (trauma, deformity, spondylolisthesis, tumor, or infection); and (7) psychiatric disorders potentially interfering with evaluation. The inclusion criteria for the non-ASD group were identical to those for the ASD group, except for the absence of prior lumbar fusion surgery. The exclusion criteria for the non-ASD group were aligned with items (3), (4), (5), (6), and (7) among the exclusion criteria for the ASD group. A total of 42 patients were ultimately enrolled in the study group. Propensity score matching was performed for patients who met the non-ASD criteria using SPSS Statistics version 27.0 (IBM Analytics, New York, USA). Based on sex, surgical level, and age (± 2 years), 42 matched patients were selected as the control group. This study was designed as an exploratory analysis, and the sample size was determined by the number of consecutive eligible patients treated during the study period. No *a priori* sample size calculation was performed. A *post hoc* power analysis based on the observed differences in VAS and ODI scores indicated that the study had sufficient power to detect clinically meaningful changes within groups.

### 2.2 Surgical procedure

**Figure 1:**
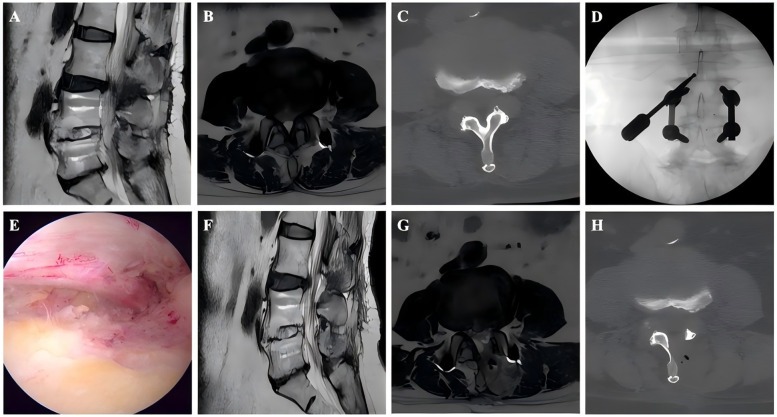
Images from an ASD patient treated with UBE. **A–B**: Preoperative MRI showed that the L4–L5 had undergone PLIF surgery previously and L3–L4 LSS. **C**: Preoperative CT. **D**: The position of the grinding drill was shown in the fluoroscopic view. **E**: The nerve was completely decompressed. **F–G**: Postoperative MRI confirmed spinal canal decompression. **H**: Postoperative CT showed a lamina defect after partial laminotomy.

**Figure 2:**
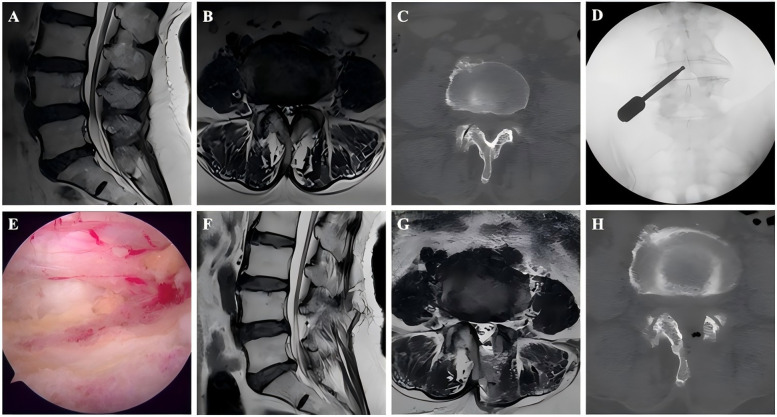
Images from a non-ASD patient treated with UBE. **A–B**: Preoperative MRI showed L4–L5 LSS. **C**: Preoperative CT. **D**: The position of the grinding drill was shown in the fluoroscopic view. **E**: The nerve was completely decompressed. **F–G**: Postoperative MRI confirmed spinal canal decompression. **H**: Postoperative CT showed a lamina defect after partial laminotomy.

Following general anesthesia with the patient in a prone position and hips flexed, C-arm fluoroscopic guidance was employed to localize the target spinal level. Two 1-cm horizontal skin incisions were created 1.5 cm lateral to the midline at the inferior endplate level of the target segment: a cranial incision for the endoscopic viewing portal connected to the light source and irrigation system and a caudal incision 1.5 cm distally as the working portal. Under endoscopic visualization, the inferior margin of the superior vertebral lamina and the superior margin of the inferior lamina were resected, and the medial aspect of the facet joint was then removed. Subsequently, the ligamentum flavum was excised to expose the dural sac and decompress the nerve. Before closing the incisions with sutures and sterile dressing application, meticulous exploration confirmed adequate neural decompression. Figures 1,2 show perioperative photographs of ASD patients and non-ASD patients, respectively.

All surgeries were performed by spine surgeons at our center who have extensive experience with UBE procedures. Prior to the start of the study, UBE surgery had already been routinely conducted at our hospital, and all participating spine surgeons had completed the required initial learning phase. The surgical steps, perioperative analgesia, anti-inflammatory treatment, and rehabilitation protocols were standardized.

### 2.3 Clinical and radiological evaluation

The data of patients in both groups, including sex, age, body mass index, smoking history, surgical level, operative time, intraoperative blood loss, postoperative hospital stay, and complications, were recorded. Low back pain (BP) and LP were assessed using the Visual Analog Scale (VAS) at the following time points: preoperatively, 3 days postoperatively, 3 months postoperatively, and 12 months postoperatively. Functional improvement was evaluated using the Oswestry Disability Index (ODI) at the same time points. Line graphs of the VAS and ODI scores were plotted according to different assessment time points. Patient satisfaction was evaluated at the final follow-up using the MacNab criteria.

All magnetic resonance imaging (MRI) scans were imported into and stored in the Picture Archiving and Communication System (PACS), with anonymized patient information. The integrated software tools within the PACS system were used to measure the cross-sectional area of the dural sac. Measurements were independently performed by two spine surgeons, and repeated 2 weeks later. The average of the two measurements was used as the final value. Both surgeons were blinded to the group allocation and were not involved in the clinical management of any patient. Intraobserver and interobserver intraclass correlation coefficients (ICC) were then calculated to determine reliability. ICC values of <0.5, 0.5–0.75, 0.75–0.9, and >0.90 were defined as indicating poor, moderate, good, and excellent reliability, respectively.

### 2.4 Statistical analysis

Statistical analysis was performed using SPSS Statistics version 27.0 (IBM Analytics, New York, USA), and figures were generated using GraphPad Prism version 10.3.1 (GraphPad Software, San Diego, California, USA). The Shapiro–Wilk test was conducted to examine the normality of continuous variables. Data with a normal distribution were analyzed using Student's *t*-test, whereas nonnormally distributed data were analyzed using the Mann–Whitney *U*-test. Categorical variables were compared using the chi-square test or Fisher's exact test. A *post-hoc* power analysis was conducted based on the observed effect size and sample size. A *P*-value < 0.05 was considered statistically significant.

## 3 Results

A total of 84 patients were enrolled in this study and divided into an ASD group (*n* = 42) and a non-ASD group (*n* = 42), with female patients accounting for 59.5% (*n* = 50). The mean age was 68.77 ± 6.81 years. Table 1 presented the baseline characteristics of the two groups. Baseline characteristics were generally comparable (Table 1), except that the operative time was significantly longer in the ASD group than in the non-ASD group (142.98 ± 34.09 vs. 105.00 ± 28.67 min, *P* < 0.001). A *post hoc* power analysis yielded an observed power of 0.83 for detecting this intergroup difference (*P* = 0.03).

**Table 1:** Baseline characteristics of included patients.

	ASD (*n* = 42)	Non-ASD (*n* = 42)	*P*-value^a^
Sex (male/female)	17/25	17/25	1.000
Age (years)	68.74 ± 6.55	68.81 ± 7.15	0.962
BMI (kg/m^2^)	26.65 ± 4.10	25.61 ± 3.89	0.239
Smoking, n	5 (11.9%)	4 (9.5%)	0.724
Surgical level			1.000
L_2_-L_3_	6 (14.3%)	6 (14.3%)	
L_3_-L_4_	8 (19.0%)	8 (19.0%)	
L_4_-L_5_	17 (40.5%)	17 (40.5%)	
L_5_-S_1_	11 (26.2%)	11 (26.2%)	
Operative time (minutes)	142.98 ± 34.09	105.00 ± 28.67	***<0**.**001***
Intraoperative blood loss (mL)	78.81 ± 53.93	70.95 ± 47.92	0.482
Postoperative hospital stay (day)	12.05 ± 2.05	11.45 ± 4.35	0.425

ASD, adjacent segment disease; BMI, body mass index.

a Bold italics indicate statistical significance.

**Figure 3:**
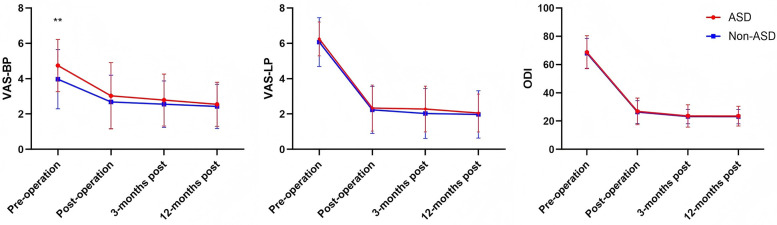
Changes in the VAS-BP, VAS-LP, and ODI scores in both groups compared with preoperative values.

Table 2 compared the preoperative and postoperative levels of BP and LP, functional outcomes, and dural sac cross-sectional area between the ASD and non-ASD groups in the cohort of patients who underwent UBE surgery. Both groups showed significant improvement in VAS-BP, VAS-LP, ODI, and dural sac cross-sectional area after surgery (Table 2). Although the ASD group had a higher preoperative VAS-BP score than the non-ASD group (4.78 ± 1.41 vs. 4.02 ± 1.65, *P* = 0.027), postoperative outcomes were comparable between groups. There was no significant difference in the postoperative dural sac cross-sectional area between the ASD and non-ASD groups (131.06 ± 50.57 vs. 117.53 ± 39.17 mm^2^, *P* = 0.174). The intraobserver and interobserver intraclass correlation coefficients for the measurement of the cross-sectional area of the dural sac were 0.92 (95% CI: 0.82–0.95) and 0.85 (95% CI: 0.78–0.91), respectively, indicating excellent and good levels of reliability. Table 3 summarizes VAS and ODI scores at different follow-up points. The ASD group had significantly higher preoperative VAS-BP than the non-ASD group (*P* = 0.027), but postoperative scores at 3 days, 3 months, and 12 months showed no statistical difference between groups. The overall trends of pain relief and functional recovery were similar. According to the MacNab criteria, the rates of excellent and good outcomes were 85.7% (36/42) in the ASD group and 88.1% (37/42) in the non-ASD group, showing no significant difference. Overall, UBE surgery provided sustained improvement in pain, function, and quality of life over the 1-year follow-up period (Figure 3).

**Table 2:** Preoperative and postoperative VAS-BP, VAS-LP, ODI scores and area of the dural sac in patients underwent UBE surgery.

	Pre-operation	Post-operation	*P*-value^a^
ASD			
VAS-BP	4.78 ± 1.41	2.99 ± 1.92	***<0*.*001***
VAS-LP	6.25 ± 0.96	2.29 ± 1.29	***<0*.*001***
ODI	68.71 ± 11.65	26.90 ± 9.48	***<0*.*001***
Area of the dural sac (mm^2^)	78.37 ± 29.89	117.53 ± 39.17	***<0*.*001***
Non-ASD			
VAS-BP	4.02 ± 1.65	2.74 ± 1.47	***<0*.*001***
VAS-LP	6.07 ± 1.38	2.23 ± 1.41	***<0*.*001***
ODI	67.98 ± 10.61	26.33 ± 8.10	***<0*.*001***
Area of the dural sac (mm^2^)	90.37 ± 40.37	131.06 ± 50.57	***<0*.*001***

VAS-BP, visual analog scale for back pain; VAS-LP, visual analog scale for leg pain; ODI, Oswestry Disability Index; ASD, adjacent segment disease; UBE, unilateral biportal endoscopy.

a Bold italics indicate statistical significance.

**Table 3:** Comparison of clinical outcomes between the ASD and non-ASD groups.

	ASD (*n* = 42)	Non-ASD (*n* = 42)	*P*-value^a^
VAS-BP			
Pre-operation	4.78 ± 1.41	4.02 ± 1.65	***0** **.** **027***
Post-operation	2.99 ± 1.92	2.74 ± 1.47	0.517
3-months post	2.74 ± 1.50	2.61 ± 1.29	0.725
12-months post	2.49 ± 1.26	2.48 ± 1.24	0.458
VAS-LP			
Pre-operation	6.25 ± 0.96	6.07 ± 1.38	0.498
Post-operation	2.29 ± 1.29	2.23 ± 1.41	0.394
3-months post	2.24 ± 1.30	2.13 ± 1.33	0.525
12-months post	2.06 ± 1.07	1.98 ± 1.34	0.758
ODI			
Pre-operation	68.71 ± 11.65	67.98 ± 10.61	0.544
Post-operation	26.90 ± 9.48	26.33 ± 8.10	0.446
3-months post	23.62 ± 7.95	23.19 ± 5.10	0.629
12-months post	23.56 ± 6.98	23.10 ± 5.12	0.830
Area of the Dural Sac (mm^2^)			
Pre-operation	78.37 ± 29.89	90.37 ± 40.37	0.125
Post-operation	117.53 ± 39.17	131.06 ± 50.57	0.174
MacNab			0.637
Excellence	11 (26.2%)	4 (9.5%)	
Good	25 (59.5%)	33 (78.6%)	
Fair	5 (11.9%)	3 (7.1%)	
Poor	1 (2.4%)	2 (4.8%)	
Complication	2 (4.76%)	1 (2.38%)	1.000
Severe complication	0	0	

VAS-BP, visual analog scale for back pain; VAS-LP, visual analog scale for leg pain; ODI, Oswestry Disability Index; ASD, adjacent segment disease.

a Bold italics indicate statistical significance.

No significant difference was found in the incidence of complications between the two groups. Two complications were recorded in the ASD group: one case of intraoperative dural tear and one case of postoperative radicular pain caused by neural edema. One complication was recorded in the non-ASD group: postoperative radicular pain secondary to neural edema. All patients recovered well after the appropriate postoperative management.

## 4 Discussion

Extending the previous fusion is the standard surgical procedure for ASD after lumbar fusion (13). However, complication rates appear to correlate positively with increased complexity of instrumentation (14). The Spine Patient Outcomes Research Trial revealed that revision surgery with extension of fusion constructs was required in nearly 10% of patients at 2-year follow-up, whereas 29% underwent additional fusion procedures (15, 16). This indicates that a substantial proportion of patients frequently require supplementary fusion surgery, which carries significant procedure-related morbidity. Furthermore, elderly ASD patients are often unwilling to undergo and unable to tolerate further fusion operations. Consequently, exploring minimally invasive alternatives that reduce trauma, accelerate recovery, and lower complication rates is clinically important.

Many studies have demonstrated the efficacy of endoscopic techniques for the treatment of ASD following spinal fusion surgery (17–20). Compared with other minimally invasive approaches, UBE allows superior instrumentation maneuverability and has an expanded surgical field of view (11). Fibrous scarring and distorted anatomy are common in ASD patients. UBE provides adequate exposure while minimizing soft tissue disruption and bony resection, thereby ensuring decompression and reducing complications (21).

Drysce et al. proposed a classification framework for treating adjacent segment degeneration based on instability and stenosis (22). According to their algorithm, isolated decompression is indicated when stenosis exists at the adjacent level without spinal instability. Building upon this paradigm, this study is the first to systematically compare UBE decompression for ASD with a matched non-ASD control group. MRI data assessment demonstrated a significant postoperative increase in the cross-sectional area of the dural sac compared with preoperative measurements. Furthermore, satisfactory clinical outcomes were achieved in most cases. Significant improvements were observed in VAS-BP, VAS-LP, and ODI scores postoperatively, which remained stable during follow-up. Additionally, the modified MacNab criteria indicated comparable effectiveness between groups, aligning with prior reports on UBE treatment for degenerative lumbar disease (9). Collectively, these findings suggest that UBE surgery effectively alleviates radicular or axial pain, reduces disability, and enhances the quality of life in elderly ASD patients.

However, ASD patients exhibited longer operative times and slightly higher intraoperative blood loss, consistent with the technical challenges inherent to revision surgery (5). Prior lumbar fusion often leads to the formation of scar tissue and altered anatomical landmarks, increasing surgical difficulty. These factors necessitate more meticulous tissue dissection, consequently prolonging the operative time. Notably, the preoperative VAS-BP score was significantly higher in the ASD group than in the non-ASD group, and remained slightly higher at all postoperative follow-up time points. This aligns with the findings reported by Cho et al. suggesting that BP symptoms may be more prevalent in ASD patients (23). We attribute this to two main factors. The ASD group presented with inherently more severe preexisting complex multilevel lumbar degeneration at the time of the index surgery. The previous fusion surgery also contributes to postoperative scar tissue formation and soft tissue adhesions, explaining why the ASD group had more pronounced preoperative BP. Second, the instrumentation permanently alters the spinal biomechanical properties. UBE surgery cannot mitigate the effect of prior fusion instrumentation on adjacent segments.

Complications represent a significant concern in ASD management. Notably, the complication rates in the ASD and non-ASD groups were 4.76% (2/42) and 2.38% (1/42), respectively, which were markedly lower than reported rates for revision lumbar fusion surgery (24). No severe complications, such as cardiopulmonary dysfunction, thrombosis, nerve root injury, massive hemorrhage, or surgical site infection, occurred in the study cohort. Furthermore, clinical evidence indicates that spinal fusion revision exacerbates segmental rigidity, triggering compensatory hypermobility at adjacent segments. This biomechanical cascade ultimately contributes to recurrent ASD and implant-related complications, such as screw loosening or rod fracture (25, 26). These biomechanical disadvantages highlight the potential advantage of decompression-only procedures such as UBE.

Notably, previous studies have suggested that the unique anatomical characteristics of the upper lumbar spine, including the relatively narrow laminar width and vertically oriented facet joints, may pose challenges to maintaining postoperative lumbar stability following UBE (27). However, in the present study, no cases of postoperative lumbar instability were observed during the 1-year-follow-up in either group. Regarding long-term stability, Kim et al. conducted a 5-year follow-up and demonstrated that UBE generally provides favorable long-term postoperative spinal stability (28). Despite these promising findings, longer-term follow-up studies are necessary to fully evaluate the enduring efficacy of UBE. Furthermore, recently proposed contralateral approaches and the “No-Punch” technique have been reported to minimize facet joint disruption, thereby contributing to the preservation of long-term lumbar stability (29–31). Given the advantages of these two techniques in maintaining spinal stability, further investigation into their surgical outcomes compared with conventional UBE in the treatment of ASD is warranted.

Considering the long-term postoperative spinal stability, patients with severe stenosis classified as Schizas grade D at our center are typically treated with UBE combined with fusion. This surgical approach differs from that used in patients with non-severe stenosis; therefore, such cases were not included in this study. For severe lumbar spinal stenosis treated with UBE decompression alone, Hu et al. reported considerable short-term clinical improvement (32). Luo et al. compared UBE decompression alone with UBE-PLIF, highlighting that both procedures achieve satisfactory short-term outcomes, while each has distinct advantages and limitations (33). The long-term spinal stability following UBE decompression alone in patients with severe stenosis, as well as the efficacy and safety of UBE combined with fusion for severe cases, could represent directions for future research.

The results of this study carry clinical significance. UBE offers an effective, minimally invasive alternative treatment for patients with adjacent segment pathology following prior fusion surgery. The minimally invasive nature of UBE helps shorten the recovery time, reduce postoperative complications, and improve patient satisfaction and functional recovery in older patients with multiple comorbidities.

Clinically, our findings indicate that UBE can serve as a feasible minimally invasive alternative for elderly or comorbid patients who may not tolerate extensive revision fusion surgery. Its less invasive nature shortens recovery, reduces perioperative risks, and maintains functional improvement.

However, this study has certain limitations. First, this study did not include a pre-study sample size or power calculation. The number of patients was determined by consecutive case enrollment, which may limit the ability to detect rare events or small between-group differences. Although a *post hoc* power analysis suggested adequate power for detecting within-group improvements in VAS and ODI, the results should still be interpreted with caution. In addition, the retrospective single-center design may introduce selection bias and limit the generalizability of the results. Second, the relatively small sample size limited our further subgroup analyses. Third, the follow-up period in this study was relatively short. With only one year of follow-up, the long-term efficacy, risk of recurrence, and durability of UBE for ASD, as well as its impact on spinal stability—particularly beyond 5–10 years—remain unknown. Thus, future prospective multicenter randomized controlled studies with larger samples and longer follow-up, systematically documenting and incorporating different subtypes of spinal stenosis are necessary to validate the long-term efficacy and safety of UBE for ASD and explore its indications and limitations in various ASD patient subgroups.

## 5 Conclusion

UBE exhibits comparable efficacy and safety in treating patients with ASD as in those with non-ASD. This technique represents a promising minimally invasive alternative for the treatment of elderly patients with stable spines, particularly elderly individuals with multiple comorbidities who cannot tolerate revision surgery. However, more prospective studies with larger sample sizes and extended follow-up periods are warranted to validate these findings.

## Data Availability

The raw data supporting the conclusions of this article will be made available by the authors, without undue reservation.
